# Cocaine/crack and cannabis use among transgender women in Goiás, Central Brazil

**DOI:** 10.1371/journal.pone.0304219

**Published:** 2024-06-06

**Authors:** Larissa Silva Magalhães, Kamila Cardoso dos Santos, Bruno Vinícius Diniz e Silva, Gabriel Francisco da Silva Filho, Grazielle Rosa da Costa e Silva, Rafael Alves Guimarães, Sandra Cristina Pillon, Karlla Antonieta Amorim Caetano, Regina Maria Bringel Martins, Megmar Aparecida dos Santos Carneiro, Robert L. Cook, Sheila Araujo Teles

**Affiliations:** 1 Faculty of Nursing, Federal University of Goiás, Goiânia, Goiás, Brazil; 2 Institute of Tropical Pathology and Public Health, Federal University of Goiás, Goiânia, Goiás, Brazil; 3 Ribeirão Preto College of Nursing, University of São Paulo, Ribeirão Preto, São Paulo, Brazil; 4 Department of Epidemiology, University of Florida, Gainesville, Florida, United States of America; University of Technology Sydney, AUSTRALIA

## Abstract

**Introduction:**

Illicit drug use is a significant public health problem. Studies have shown a high prevalence of cocaine and cannabis use in transgender women (TGW).

**Objective:**

To describe the consumption patterns of cannabis and cocaine/crack use and variables associated with their use in TGW in Central Brazil.

**Methods:**

A cross-sectional study was conducted on TGW in Goiás, Brazil. Participants were recruited using a respondent-driven sampling method and were interviewed face-to-face about cannabis and crack-cocaine and the variables associated with them. The Alcohol Smoking and Substance Involvement Screening Test was used to assess substance use. Unweighted logistic regression was used to identify variables associated with cannabis and crack cocaine use. *P*-values < 0.05 were considered statistically significant.

**Results:**

A total of 440 transgender women participated in the study. Their median age was 25 years (interquartile range: 20.5–29.5 years). Most participants were single (85.5%) and had engaged in sex work in their lifetime (58.6%). Cannabis was reported by 68.9% and 53.4% of participants in their lifetime and in the past three months, respectively, and cocaine/crack use was reported by 59.8% and 44.1% of participants in their lifetime and the past three months, respectively. Of the participants, 10.2% reported high-risk cannabis use, and 9.1% reported high-risk cocaine/crack use. Furthermore, 35% of participants reported using both drugs. Previous physical violence (Adjusted Odds Ratio (AOR): 2.37), inconsistent condom uses during anal sex (AOR: 2.17), and moderate-/high-risk cocaine/crack use (AOR: 3.14) were associated with high-risk cannabis use. Previous sexual violence (AOR: 2.84), previous STI (AOR: 2.90), moderate-/high-risk cannabis (AOR: 3.82), and binge drinking (AOR; 3.28) were associated with high-risk cocaine/crack use.

**Conclusion:**

Our study found a high frequency, significant overlap in the use of cannabis and cocaine/crack use and violence associated with these drugs consumption among TGW, highlighting the urgent need for health policies for drug disorders among this socially marginalized group.

## Introduction

Illicit substance use is an important public health problem. Globally, in 2020, an estimated 284 million people aged 15–64 years had used a drug within the past 12 months [1]. This represents an increase of 26% compared to 2010. Cannabis remains a major drug used (209 million), followed by opioids (61 million), amphetamines (34 million), cocaine (21 million), and “ecstasy” (20 million). Drug use can have negative consequences, such as physical and mental health disorders (mainly addiction), human immunodeficiency virus (HIV) infection, liver diseases related to hepatitis, overdose, and premature death [1]. According to the Global Burden of Disease study, 494,000 drug-related deaths occurred in 2019 [2].

In South and Central America, cannabis and cocaine are the major drugs consumed, and cocaine use is the primary reason for seeking treatment for drug use disorders [1]. In 2017, the Third National Survey on Drug Use in the Brazilian Population estimated that the lifetime and in the past 12 months prevalence of drug use was 7.7% and 2.5%, respectively, for cannabis use, 3.1% and 0.9%, respectively, for cocaine use, and 0.9% and 0.3%, respectively, for crack and similar drugs [3].

Some studies have shown that gender minorities, such as transgender women (TGW) use more drugs than the general population [4]. These individuals experience discrimination, prejudice, and several forms of violence that, in turn, reduce their opportunities for education and employment and increase their chances of drug use [5, 6].

A study conducted among TGW in Chicago, Houston, and Los Angeles, USA, found a prevalence of cannabis and cocaine/crack use of 81% and 29%, respectively [7]. Another study conducted among TGW in Los Angeles, USA, found a prevalence of cannabis use of 26.7% [8], and a study conducted among TGW in California, USA, found a prevalence of cocaine/crack use of 6.3% [9]. Furthermore, some studies have shown that TGW are more likely to participate in sexual intercourse while under the influence of illicit drugs than other sexual minorities, which may increase their risk of acquiring HIV and other STI [10, 11].

Most studies on drug use among TGW have been conducted in the USA. Few studies have been conducted on drug use patterns in this population in the Global South, including regions with drug-trafficking routes, such as Central Brazil [12]. Therefore, to contribute to the knowledge about drug use among TGW and assist stakeholders in making decisions about public health policies, we conducted a study on the prevalence and consumption patterns of cocaine/crack and cannabis use, the main drugs used worldwide and in Brazil [1, 3], among TGW. The study aimed to estimate the prevalence of substance use in TGW in Goiás, Central Brazil, and to analyze the factors associated with high-risk polydrug (cocaine/crack and cannabis use).

## Methods

### Study design

This cross-sectional study was conducted between April 2018 and November 2019 among TGW in Goiás, Central Brazil.

### Study population and sample size

**Fig 1:**
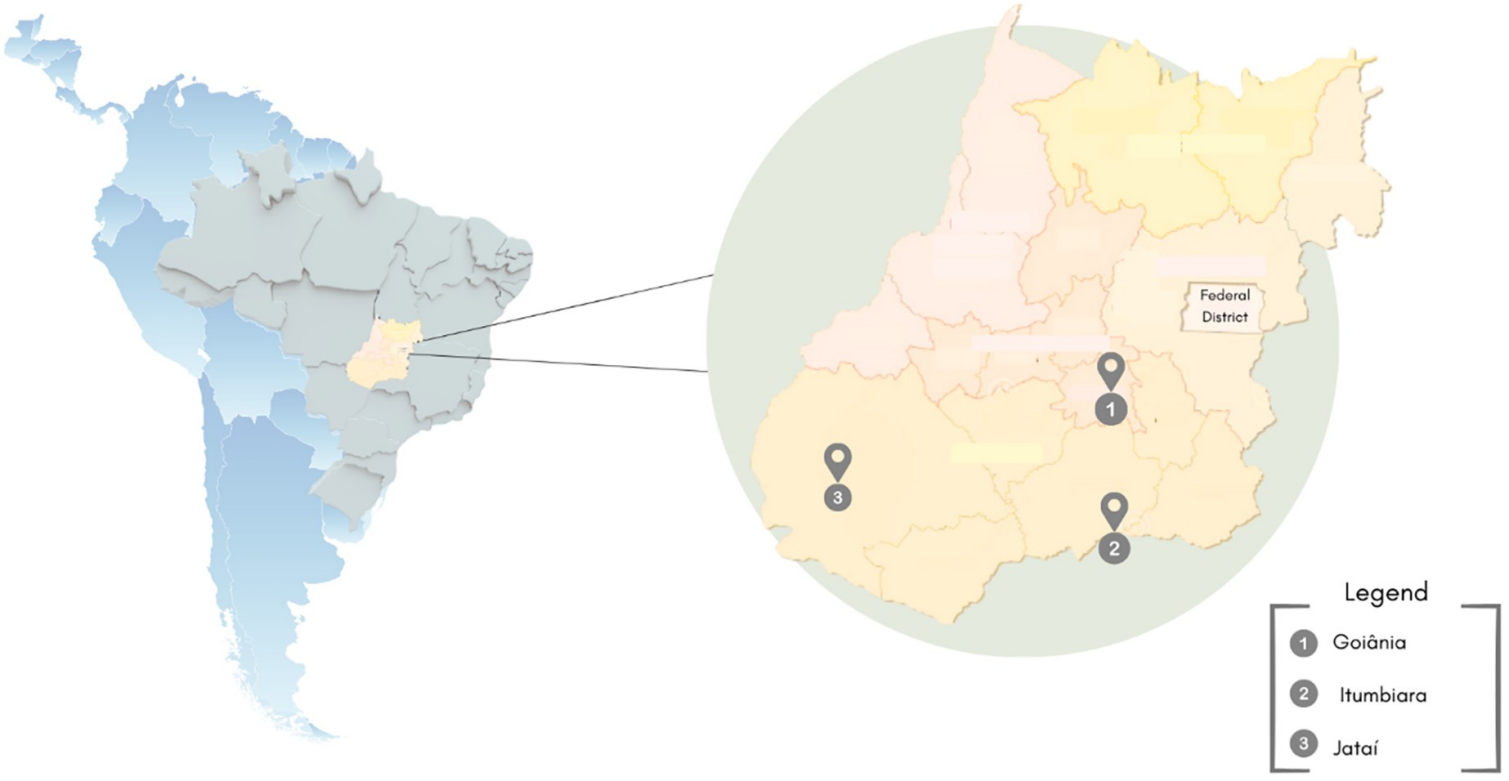
Geographical location of the municipality of Goiás, Central Brazil. This study was conducted in three cities: Goiânia (population: 1,437,366; Human Development Index [HDI]: 0.799), Itumbiara (population: 107,970; HDI: 0.752), and Jataí (population: 105,729; HDI: 0.757) [13, 14].

Brazil is considered an important drug-trafficking route in South America, and Goiás (Fig 1) is one of the states with the greatest number of cocaine and cannabis seizures by the Federal Road Police [1].

Transgender is an umbrella term used to define persons whose gender assigned at birth differs from their identity, expression, or behavior [15]. For this study, the required sample size was estimated as 406 participants, assuming a prevalence of cocaine drug use of 5% [16], a significance level of 95% (α = 0.05), a precision of 3%, and a design effect of 2.0.

Individuals who defined themselves as TGW to the study recruiter at enrollment and presented a valid RDS coupon were eligible for the study. During the interviews, an individual was found to be noticeably under the influence of psychoactive drugs (drunk or incoherent) and was excluded from this study.

Drug use was defined according to the United Nations Office on Drugs and Crime definition [1] as *substances controlled under international drug control conventions and their non-medical use*.

### Data collection

RDS has been widely used in studies of hard-to-reach populations such as TGW [17, 18]. For this study, we used data from a bio-behavioral study using respondent-driven sampling (RDS) method among TGW in Goiás, Central Brazil. Details were described elsewhere [19].

Initially, eight individuals with diverse ages, education, and jobs who self-identified as TGW were purposely recruited based on their interest in participating in the study, their leadership, and their high popularity in the TGW communities. They were denominated “seeds” and started the recruitment chains. For this purpose, they received three recruitments unique RDS coupons to invite other TGW of their social network, and one reward coupon. It was defined that each participant would receive 10 BRL as allowance/incentive and an additional 10 BRL for each recruited member who also accepted to participate. A total of 1312 invitation were distributed by eight seeds, and 440 individuals participated in the study.

Participants recruited by seeds who self-defined as transgender women and presented a valid RDS coupon were enrolled in the study. Persons who were found to be noticeably under the effects of psychoactive drugs (drunk or incoherent) were excluded. The recruitment started in April 2018 and ended in October 2019.

All participants provided written informed consent. The Research Ethics Committee waived the need for consent from parents and guardians of minors. They were interviewed by a research team member previously trained for the study purpose. All interviews were face-to-face using a structured questionnaire based on the *Diversidade e Valorização da Saúde* [Diversity and Valuing Health] (DIVaS) study [20].

### Measurements and independent variables

Prevalence (%) of cannabis and cocaine/crack use in lifetime: (number of individuals who use cannabis in lifetime/number of individuals interviewed) *100. We used the first question of the Alcohol, Smoking, and Substance Involvement Screening Test (ASSIST).

Prevalence (%) of cannabis use past in the three months: (number of individuals who reported cannabis use past three months/number of total individuals interviewed) *100. We used the second question of ASSIST.

Prevalence (%) of cocaine/crack use in the past three months: (number of individuals who reported cocaine/crack use in past three months/number of total individuals interviewed) *100. We used the second question of ASSIST.

Prevalence (%) of cannabis use and cocaine/crack use in the past three months: (number of individuals who reported cannabis and cocaine/crack use in the past three months/number of total individuals interviewed) *100. We used the second question of ASSIST.

The proportion (%) of cannabis and cocaine/crack consumption patterns involved creating a variable by summing the scores from the ASSIST (Question 2 to Question 7), subsequently stratified into three categories: “0” low-risk, “1” moderate-risk use, “2” high-risk use.

#### Sociodemographic data

The sociodemographic variables included age (continuous variable; categorized according to the median age), marital status single (yes/no), race/ethnicity (classified as white, and non-white [mixed-race, black, Asian, and Indigenous], monthly income (continuous variable) and Education categorized according to the Brazilian education grade: less than Primary level (< 9 of education), complete primary level or incomplete secondary level (9–11 years of education), and full secondary level (12 years of schooling or over).

#### Sexual behavior

Sexual behavior questions included the number of sexual partners in the past seven days (continuous variable; categorized according to quartiles); sex with drug-using partners (“Have you ever had sexual intercourse with a partner who uses drugs?” yes/no); age at first intercourse (“How old were you when you had your first sexual intercourse?” continuous variable; previous sexually transmitted infections (STIs) (“Have you ever had an STI?” yes/no), and sexual intercourse with men and women (“In the past six months, have you had sex with men and women?” yes/no); sex work in a lifetime (yes/no).

#### Other variables

Physical violence (Have you ever been physically assaulted in your life?” yes/no); sexual violence (“Have you ever been physically forced to have sex in your life?” yes/no); previous prison (“Have you ever been arrested?” yes/no); binge drinking (defined as ingesting four or more alcoholic beverages on one occasion) (yes/no).

### Dependent variables

For this study, the outcomes were high-risk use of cocaine/crack and high-risk use of cannabis (ASSIST score ≥27), categorized dichotomously into “0” No and “1” Yes. ASSIST has a standardized structure, is quick to administer, allows the approach to several classes of subjects, is easy to interpret, and can be used by health professionals with different backgrounds. This instrument has been validated in Brazil and has good sensitivity, specificity, internal consistency, and validity, suggesting its usefulness in detecting psychoactive substance abuse [21]. It has eight questions that evaluate lifetime use, use in the past three months, and the pattern of use of nine substance groups (alcohol, tobacco, cannabis, cocaine, amphetamines, inhalants, sedatives, hallucinogens, and opioids). For each substance, the test provides a score indicating low (0–3), moderate (4–26), or high-risk use (≥27) [22].

### Data analysis

The data were collected and entered in a database using EpiData version 3.1 (EpiData Association, Odense, Denmark) and exported to SPSS, version 28.0 (IBM Corp., Armonk, NY, USA) for analysis. Prevalence unadjusted by RDS with 95% confidence intervals (CIs) was estimated for all categorical variables.

Multivariable logistic regression models with a forward selection algorithm were used to assess the adjusted associations of variables that showed p < 0.20 in the bivariate analysis. The adjusted odds ratio and 95% CIs were calculated, and variables in the final model with a p-value of ≤ 0.05 were considered statistically significant. The Hosmer-Lemeshow test was used to assess the goodness of fit of the logistic regression model [23].

The study was approved by the Research Ethics Committee of the *Universidade Federal de Goiás*-UFG (Protocol Number 2,358,818).

## Results

A total of 440 TGW participants participated in this study. Among them, cannabis use ever and in the past three months was reported by 68.9% and 53.4%, respectively, and cocaine/crack use ever and in the past three months was reported by 59.8% and 44.1%, respectively. Table 1 shows the prevalence of cannabis and cocaine/crack use among participants. Moderate risk was the most prevalent pattern of cannabis and cocaine/crack use. Of the participants, 10.2% reported high-risk cannabis use, and 9.1% reported high-risk cocaine/crack use.

**Table 1:** Patterns of cannabis and cocaine/crack use among 440 transgender women in *Goiás–GO*, 2018–2019.

Patterns of drug use	n	%	95% CI*
**Patterns of Cannabis Use**			
Use in lifetime	303	68.9	64.4–73
Use past in the three months	235	53.4	48.7–58
Low-risk	13	3.0	1.73–4.99
Moderate-risk use	177	40.2	35.7–44.8
High-risk use	45	10.2	7.7–13.4
**Patterns of Cocaine use**			
Use in lifetime	263	59.8	55.1–64.3
Use in the past three months	194	44.1	39.5–48.8
Low-risk use	27	6.1	4.25–8.78
Moderate-risk use	127	28.9	24.8–33.3
High-risk use	40	9.1	6.8–12.1
Cannabis and cocaine/crack use in the past three months	154	35	30.7–39.5

^a^Drug use was measured using the Alcohol, Smoking, and Substance Involvement Screening Test (ASSIST).

*95%CI: 95% Confidence Interval.

Most participants were young adults, non-white, with a low income, low level of education, and engaged in sex work (Table 2). They had their first sexual intercourse at a young age (median: 13 years), and many participants had experienced physical and sexual violence. Half of the participants reported a previous STI, and participants had a median of 10 sexual partners in the past seven days. Approximately one-third of participants had sex with both men and women and used condoms during sexual intercourse. Almost all participants had drug users as sexual partners. Of the participants, 23.9% reported spending time in prison, and 56.6% reported binge drinking.

**Table 2:** Factors associated with high-risk cannabis use among transgender women in *Goiás*, *Central Brazil–GO*.

Variables	High-risk cannabis use^a^			
	Yes	No	OR (95% CI*)	*p*-value
	n (%)	n (%)		
**Age (years), median (IQR)**	24 (5)	25 (10)	0.96 (0.92–1.01)	0.110
**Monthly income (BLR)**,^c^ **median (IQR)**	3,000 (3,00)	2,000 (2,000)	1.00 (1.00–1.00)	0.032
**White race, n (%)**				
Yes	6 (6.7)	84 (93.3)		
No	39 (11.2)	309 (88.8)	1.77 (0.72–4.31)	0.211
**Level of education, n (%)**				
Secondary completed or higher	14 (6.9)	188 (93.1)		
Primary completed, secondary incomplete	22 (13.9)	1136 (86.1)	2.17 (1.07–4.40)	0.031
Less than primary	9 (11.3)	71 (88.8)	1.70 (0.71–4.11)	0.237
**Sex worker, n (%)**				
No	9 (4.9)	173 (95.1)		
Yes	36 (14.0)	222 (86.0)	3.12 (1.46–6.45)	0.003
**Age (years) at 1**^**st**^ **sexual intercourse, median (IQR)**	13 (4)	13 (4)	0.95 (0.86–1.04)	0.274
**Previous sexual violence, n (%)**				
No	16 (7.0)	211 (93.0)		
Yes	29 (13.7)	182 (86.3)	2.10 (1.11–3.99)	0.023
**Previous physical violence, n (%)**				
No	18 (6.9)	243 (93.1)		
Yes	27 (15.3)	150 (84.7)	2.43 (1.29–4.56)	0.006
**Previous STI (lifetime), n (%)**				
No	15 (6.8)	205 (93.2)		
Yes	30 (13.6)	190 (86.4)	2.16 (1.13–4.13)	0.020
**Number of sexual partners in past seven days, median (IQR)**	18 (22)	10 (27)	1.01 (1.00–1.01)	0.267
**Sex with men and women, n (%)**				
No	22 (7.4)	275 (92.6)		
Yes	23 (16.5)	116 (83.5)	2.48 (1.33–4.62)	0.004
**Condom use during anal sex, n (%)**				
Always	10 (6.4)	146 (93.6)		
Sometimes/never	35 (12.7)	240 (87.3)	2.13 (1.02–4.43)	0.043
**Sexual partner drug user, n (%)**				
No	-	56 (100,0)		
Yes	45 (11.9)	332 (88.1)	-	
**Previous prison, n (%)**				
No	28 (8.5)	303 (91.5)		
Yes	17 (16.3)	87 (83.7)	2.11 (1.11–4.04)	0.024
**Moderate-/high-risk cocaine/crack, n (%)**				
No	14 (5.1)	259 (94.9)		
Yes	31 (18.6)	136 (81.4)	4.22 (2.17–8.19)	<0.001
**Binge alcohol drink, n (%)**				
No	12 (6.3)	179 (93.7)		
Yes	33 (13.3)	216 (86.7)	2.28 (1.14–4.54)	0.019

^a^High-risk use was defined as a score of ≥27 using the Alcohol, Smoking, and Substance Involvement Screening Test (ASSIST).

^b^Only valid data.

^c^During the study period, 1 US$ was equivalent to 3.91 BRL.

*95% CI, 95% confidence interval; IQR, interquartile range; OR, odds ratio

Tables 2 and 3 show variables associated with high-risk cannabis and high-risk cocaine use, respectively. The forward multivariable logistic regression models included those that presented *p* values < 0.20. The final models identified three variables independently associated with high-risk cannabis use among TGW: physical violence (adjusted odds ratio [AOR]: 2.37; 95% CI:1.22–4.62), inconsistent condom use during anal sex (AOR: 2.17, 95% CI: 1.00–4.69), and moderate-/high-risk cocaine/crack (AOR: 3.14; 95% CI: 1.57–6.28). The variables sexual violence (AOR: 2.84; 95 CI: 1.26–6.42), previous STI (AOR: 2.90; 95% CI: 1.31–6.38), moderate-/high-risk cannabis use (AOR: 3.82; 95% CI: 1.49–9.79), and binge drinking (AOR: 3.28; 95% CI: 1.39–7.75) were independently associated with high-risk cocaine use (Table 4).

**Table 3:** Factors associated with high-risk cocaine/crack use among transgender women in *Goiás*, *Central Brazil*.

	High-risk cocaine/crack^a^			
Variable	Yes	No		
	n (%)	n (%)	OR (95% CI*)	*p*-value
**Age (years), median (IQR)**	25 (5)	25 (9)	0.98 (0.94–1.02)	0.373
**Monthly income (BRL)**,^c^ **median (IQR)**	3,000 (2,500)	2,000 (2,000)	1.00 (1.00–1.00)	0.215
**White race, n (%)**				
Yes	7 (7.8)	83 (92.2)		
No	33 (9.5)	315 (90.5)	1.24 (0.53–2.91)	0.617
**Level of education, n (%)**				
Secondary completed or higher	16 (7.9)	186 (92.1)		
Primary completed, secondary incomplete	3 (8.2)	145 (91.8)	1.04 (0.49–2.24)	0.915
Less than primary	11 (13.8)	69 (86.3)	1.85 (0.82–4.19)	0.138
**Sex worker, n (%)**				
No	5 (2.7)	177 (97.3)		
Yes	35 (13.6)	223 (86.4)	5.56 (2.13–14.76)	< 0.001
**Age (years) at 1**^**st**^ **sexual intercourse, median (IQR)**	12.5 (4)	13 (4)	0.92 (0.84–1.02)	0.119
**Previous sexual violence, n (%)**				
No	10 (4.4)	217 (95.6)		
Yes	30 (14.2)	181 (85.8)	3.60 (1.71–7.56)	0.001
**Previous physical violence, n (%)**				
No	15 (5.7)	246 (94.3)		
Yes	25 (14.1)	152 (85.9)	2.70 (1.38–5.28)	0.004
**Previous STI (lifetime), n (%)**				
No	11 (5.0)	209 (95.0)		
Yes	29 (13.2)	191 (86.8)	2.88 (1.40–5.93)	0.004
**Number of sexual partners in past 7 days, median (IQR)**	19 (20)	10 (27)	1.00 (1.00–1.01)	0.016
**Sex with men and women, n (%)**				
No	18 (6.1)	279 (93.9)		
Yes	22 (15.8)	117 (84.2)	2.91 (1.51–5.63)	0.001
**Condom use during anal sex, n (%)**				
Always	9 (5.8)	147 (94.2)		
Sometimes/never	31 (11.3)	244 (88.7)	2.07 (0.96–4.48)	0.063
**Sexual partner drug user, n (%)**				
No	-	56 (100.0)		
Yes	40 (10.0)	337 (89.4)		
**Previous prison, n (%)**				
No	22 (6.6)	309 (93.4)		
Yes	18 (17.3)	86 (82.7)	2.94 (1.51–5.73)	0.002
**Moderate-/high-risk cannabis use, n (%)**				
No	6 (2.8)	212 (97.2)		
Yes	34 (15.3)	188 (84.7)	6.39 (2.62–15.56)	< 0.001
**Binge alcohol drink, n (%)**				
No	8 (4.2)	183 (95.8)		
Yes	32 (12.9)	217 (87.1)	3.37 (1.52–7.50)	0.003

^a^High-risk was defined as a score of ≥27 on the Alcohol, Smoking, and Substance Involvement Screening Test (ASSIST).

^b^Only valid data.

^c^During the study period, 1 US$ was equivalent to 3.91BRL.

*95% CI, 95% confidence interval IQR, interquartile range; OR, odds ratio

**Table 4:** Analysis of factors associated with high-risk cannabis and cocaine/crack use among transgender women in *Goiás*, *Central Brazil–GO*, 2018–2019.

Variable	High-risk cannabis use^a^		High-risk cocaine/crack use^a^	
	AOR^c^ (95% CI) ^d^	p-value	AOR^e^ (95% CI)	p-value
**Previous sexual violence**			**2.84 (1.26–6.42)**	**0.012**
**Previous physical violence**	**2.37 (1.22–4.62)**	**0.011**	1.98 (0.93–5.21)	0.076
**Previous STI (lifetime)**	1.87 (0.94–3.69)	0.073	**2.90 (1.31–6.38)**	**0.008**
**Sex worker**			2.43 (0.86–6.88)	0.093
**Sexual practice with men and women**	1.88 (0.97–3.65)	0.063	1.81 (0.88–3.76)	0.108
**Inconsistent condom use during anal sex**	**2.17 (1.00–4.69)**	**0.050**		
**Moderate** ^**b**^ **-/high** ^**a**^ **-risk cannabis use**			**3.82 (1.49–9.79)**	**0.005**
**Moderate** ^**b**^ **-/high** ^**a**^ **-risk cocaine/crack use**	**3.14 (1.57–6.28)**	**0.001**		
**Binge drinking**			**3.28 (1.39–7.75)**	**0.007**

^a^High-risk use was defined as a score of ≥27 using the Alcohol, Smoking, and Substance Involvement Screening Test (ASSIST).

^b^Moderate-use risk was defined as a score of 4–26 using the Alcohol, Smoking, and Substance Involvement Screening Test (ASSIST).

^c^AOR, adjusted odds ratio by monthly income, level of education, sex work, previous sexual violence, previous physical violence, previous STI, sex with men and women, condom use during anal sex, previous prison, moderate-/high-risk cocaine/crack use, binge drinking.

^d^CI, confidence interval.

^e^AOR by level of education, sex work, age at 1^st^ sexual intercourse, previous sexual violence, previous physical violence, previous STI, number of sexual partners in past 7 days, number of sexual partners in past 7 days, previous prison, moderate-/high-risk cannabis use, binge drinking.

^d^Hosmer–Lemershow test (p = 0.308); ^e^Hosmer–Lemershow test (p = 0.761)

## Discussion

We found a high prevalence of cannabis and cocaine use among TGW in Central Brazil, a vulnerable population whose lives are affected by violence, discrimination, and prejudice. In our study, a higher proportion of risky behaviors was found among those who reported cannabis and cocaine/crack use than among those who did not, and a significant synergism was found between these drugs.

According to the Third National Survey on Drug Abuse in the Brazilian Population 2017, 2.5% of persons 12–65 years of age used cannabis in the past 12 months. Regarding powder cocaine and crack, the prevalence was 0.9% and 0.3%, respectively [3]. This prevalence is much lower than that found among the TGW in this study: 68.9% for cannabis and 59.8% for cocaine/crack. Furthermore, 40.2% and 10.2% of participants showed moderate- and high-risk cannabis use, and 28.9% and 9.1% showed moderate- and high-risk cocaine/crack use, respectively, indicating that this population has a high risk of developing substance use disorder.

Notably, one-third of TGW reported using both cocaine/crack and cannabis. In the multivariable logistic regression analysis, they were associated with each other, highlighting the overlap of these drugs. A systematic review and meta-analysis of 16 studies from four countries found that 64% of cocaine users also used cannabis [24].

Although the effects of the concomitant use of cocaine and cannabis are still unclear, for some drug users, the overstimulation caused by cocaine is offset by concomitant cannabis use due to its relaxing effects. Participants in a qualitative study reported that cannabis decreases the stimulatory effects of cocaine, improves sleep and appetite, and reduces cravings [25]. Despite these “benefits” of co-use cocaine and cannabis, Wheelers et al. [26] found a decreasing total sleep time in individuals who used cocaine and cannabis compared with those who used cocaine only, and Oliveira Junior et al. [27] found that individuals who used cocaine and cannabis had worse processing speed, inhibitory control, and sustained attention than those who used cocaine only. Furthermore, some studies have found an increased risk of cocaine relapse and severe cocaine withdrawal among cannabis users [28, 29].

Although in the bivariate analysis, binge alcohol consumption was associated with both high-risk cannabis and cocaine use, in the multivariable logistic regression analysis, this variable remained independently associated only with high-risk cocaine use. A meta-analysis also showed a high frequency of concurrent/simultaneous alcohol and cocaine use and alcohol and cannabis use. However, the prevalence of concurrent/simultaneous alcohol and cocaine use was higher than that of concurrent/simultaneous cannabis and cocaine use [24]. Notably, alcohol and cocaine form the active metabolite, cocaethylene, a transesterification product of both drugs. This metabolite is as potent as cocaine and has a longer elimination half-life, prolonging the drug effect [30, 31]. Therefore, we could speculate that this potentiation of cocaine contributes to concurrent/simultaneous alcohol use.

The findings of a higher prevalence of high-risk drug use among those who reported sex work, previous STIs, and inconsistent condom use are consistent with the findings of previous studies [32–34]. Substance use disorder has a negative effect on the control of STIs by interfering with the user’s decision-making and favoring sexual practices with a higher risk of acquiring STIs. Drug use can interfere with adherence to HIV pre-exposure prophylaxis [35], contributing to the burden of HIV [36].

Notably, none of the participants in this study who denied having drug-using sexual partners were identified as having high-risk drug use. This finding is consistent with the findings of previous studies that some power relationships can interact with the partner’s drug use status [37, 38].

A survey carried out in 2015 among transgender people in the United States showed 46% of the respondents experienced verbal harassment and 9% physical attack because of being transgender in the past year, and nearly 47% have been sexually assaulted in their lifetime [39].

In this study, 48.2% of participants reported experiencing sexual violence and 40.4% reported experiencing physical violence. These findings align with Brazil’s history of killings of transgender people [40]. Furthermore, sexual violence was associated with high-risk cocaine/crack use, whereas physical violence was associated with both high-risk cocaine/crack and high-risk cannabis use. Although our study design prevents the inference of causality, these findings suggest that experiences of violence increase the risk of drug abuse, which can be understood as a coping mechanism for dealing with trauma [10, 41].

This study has some limitations. First, reverse causality should not be disregarded because of the cross-sectional study design. Second, the use of self-reported measures and behavioral variables may have led to social desirability bias. We explored these potential biases and attempted to minimize their effects by using validated instruments in the data collection and training of the interviewers. Although RDS is an effective method for recruiting hard-to-reach individuals, such as TGW, there is still non-consensus on the more appropriate method for data analysis of RDS samples: weighted regression or unweighted regression [42]. Therefore, as with other authors, we did not incorporate adjustments for potential recruitment biases in RDS methodology in the analyses [43, 44]. Therefore, the estimates of the prevalence of high-risk drug use may not be representative of TGW in Central Brazil, and our results should be interpreted with caution.

Despite these limitations and the significant challenge of connecting with a hard-to-reach population, this study contributes to existing literature in several ways. These results provide information on substance use in Goiás. They allowed us to establish a set of factors associated with substance use among TGW, contributing to better planning and execution of strategies, and formulating and implementing policies and programs to address the health service needs of this population.

In conclusion, this study identified a high prevalence of high-risk cannabis and cocaine/crack use among TGW living in three cities along a drug-trafficking route in Central Brazil, suggesting that drug use is associated with the vulnerabilities experienced by this population. Therefore, multi-level interventions and resources are required to increase the availability of treatment and access to health services.

## Supporting information

S1 Data (XLS)
